# Flax/PP and Flax/PLA Thermoplastic Composites: Influence of Fire Retardants on the Individual Components

**DOI:** 10.3390/polym12112452

**Published:** 2020-10-23

**Authors:** Baljinder K. Kandola, Wiwat Pornwannachai, John Russell Ebdon

**Affiliations:** 1 Institute for Materials Research and Innovation, University of Bolton, Deane Road, Bolton BL3 5AB, UK; wiwatpor@scg.com (W.P.); J.Ebdon@bolton.ac.uk (J.R.E.); 2 SCG Chemicals Co., Ltd., 1 Siam Cement Rd., Bang Sue, Bangkok 10800, Thailand

**Keywords:** natural fibre-reinforced composites, polypropylene, polylactic acid, flax fibre, fire retardants, pyrolysis combustion flow calorimetry, cone calorimetry

## Abstract

This study is based on previously reported reaction to fire properties of flax fibre-reinforced polymeric (polypropylene, PP and polylactic acid, PLA) composites, prepared by pre-treating the fabrics with different fire retardants (FRs) prior to composite preparation. It was observed that while all of these treatments were very effective in flax/PLA in terms of achieving a V-0 rating in a UL-94 test, only an organophosphonate FR was capable of achieving a V-0 rating for flax/PP. However, all fire-retardant treatments impaired the mechanical properties of the composites; the reduction was more in flax/PLA compared to flax/PP composites. To understand these effects further, here thermal analysis and pyrolysis combustion flow calorimetry of the composites and each component separately treated with FRs have been conducted and the results analysed in terms of the effect on each component so as to observe any interaction between the different components. The results indicated that in flax/PLA composites, the water released during FR catalysed dehydration-decomposition of flax may hydrolyse PLA, changing decomposition pathway of PLA to produce less flammable volatile, hence resulting in reduced flammability.

## 1. Introduction

Natural fibre composites gained popularity after being taken up by the automotive industry about 25 years ago, although reports of their first usage date back to the 1940s [1]. Despite this there has been limited investigation of fire retardancy in these materials until about ten years ago, mainly because fire regulations are less stringent for the automotive sector than for some other areas [2]. However, as applications of natural fibre composites are becoming more widespread, fire performance is also becoming more important, the major concern being that as opposed to non-flammable glass/carbon fibres, natural fibres are flammable and may add to the flammability of the polymeric matrix. However, it has been shown that inclusion of even natural fibres reduces the flammability of the composites as compared to that of a neat polymer matrix of similar thickness [3]; the reduction though is less than that for glass/carbon fibre inclusions. This is because despite the flammable nature of natural fibres such as flax, jute, etc., their charring tendency helps in reducing the propensity for burning of a composite compared to the neat polymer; i.e., the time-to-ignition (TTI), peak heat release rate (PHRR) in a cone test and rate of burning is reduced, whereas the duration of burning is increased [4,5,6]. These effects increase with increasing fibre content [7]. The charring property of cellulosic fibres can be further enhanced by using phosphorus and nitrogen based, condensed-phase active fire retardants, which are well established for cotton textiles [8]. Ideally, in natural fibre composites, effective fire retardants would be those that are effective on both matrix and the fibre, which is challenging owing to different chemical structures of the two polymers. The fire-retardant chemicals though should not be reactive with the fibre during the processing stage otherwise the mechanical properties will be affected. While the ligno-cellulosic derived natural fibres can be easily fire retarded using both vapour-phase (halogenated) and condensed-phase active fire retardants (FRs), the latter are not very effective in non-oxygen containing polymers such as polypropylene. Vapour-phase active organohalogen FRs are effective in all polymer types, but due to environmental issues associated with their usage, are not a popular choice.

The choice of fire retardant and method of application also depend on the manufacturing process used for composite preparation. For example, if the thermoplastic polymer is used in sheet form or even as pellets, fire retardants would be added to the polymer by melt blending prior to making composites [5,9,10]. However, if fabrics from co-woven or commingled natural and synthetic fibres are used for composite fabrication by melt pressing stacked textile layers, the easiest method of fire retardation is to apply aqueous fire retardant solutions using a traditional pad/dry technique, as reported previously [11,12]. The application of four commercially available fire-retardant formulations to fabrics from commingled flax/polypropylene and flax/polylactic acid prior to melt pressing to make composites was carried out in work reported previously [11,12]. The FR formulations were commercial products containing, as active FR chemicals: (i) ammonium sulfamate (ii) ammonium bromide, (iii) guanidine dihydrogen phosphate, an organophosphate, and (iv) guanylurea methyl phosphonate, an organophosphonate. It was observed that all FRs were more effective in polylactic acid (PLA) than in polypropylene (PP) composites. With all FRs, flax/PLA achieved a V-0 rating in a UL-94 test, whereas for flax/PP only the organophosphonate FR was capable of imparting a V-0 rating. In all cases though, flammability was reduced as measured by cone calorimetry. However, all fire retardants impaired the mechanical properties of composites, and the reduction was more pronounced in flax/PLA than in flax/PP. However, this is understandable in that PP is non-reactive, hence FRs are less effective in flax/PP composites, whereas, although PLA is a linear polyester and hence chemically should not be reactive towards these FRs at room temperature, there is a possibility of reaction with –OH and –COOH end groups at high temperatures. In order to understand the effects of FRs on different components of the composites and to explore any interaction between their pyrolysis products, PP and PLA fibres have been individually treated with these FRs and then the thermal decomposition and flammability of all the treated components and composites evaluated using thermal analysis and pyrolysis flow calorimetry (PCFC), respectively. PCFC as opposed to cone calorimetry has been used as an indicator of flammability or fire retardancy of composites as well as of individual components. The rationale for this is that for cone calorimetric tests, samples of similar geometry must be used for all components of the composites, and in this case, while plaques of similar dimensions as the composites can be prepared from unmodified and fire retarded PP and PLA, flax fibres/fabrics cannot be melt pressed into plaques. It is well known that all conventional FR tests are geometry dependent, and so PCFC, which uses mg-sized samples [13], was used. Based on PCFC measurements, the actions of different FRs on the different components have been analysed in detail. These results have also been used to determine whether or not PCFC can be used as an indicator of the combustibility of composites by comparing the results of PCFC with those from cone calorimetry.

## 2. Materials and Methods

### 2.1. Materials

#### 2.1.1. Fabrics and Fibres

Plain woven fabrics (4 × 4 plain weave structure) were sourced from Composites Evolution, Chesterfield, UK:

1. Flax woven fabric; 467 g/m^2^ area density
2. Commingled flax/PP woven fabric (50/50 wt-%); 465 g/m^2^ area density
3. Commingled flax/PLA woven fabric (50/50 wt-%); 493 g/m^2^ area density

Short staple fibres of polypropylene (average fibre length 60 mm, linear density 2.2 dTex) and polylactic acid (average fibre length 75 mm, linear density 3.3 dTex) were sourced from Tilsatec Advanced Textile Materials, Wakefield, UK.

#### 2.1.2. Fire Retardants (FRs)

**Scheme 1:**
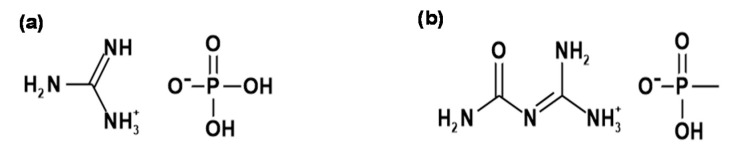
The chemical structures of (**a**) guanidine dihydrogenphosphate (GDP) and (**b**) guanylurea methylphosphonate (GUP).

Four commercially available, water-soluble, fire retardants, designed for cellulosic fibres were used. Owing to the commercial sensitivity of the work, the source of fire retardant formulations has not been provided and these have been identified hereby only by the active FR components they contain: (i) ammonium sulfamate (AS), (ii) ammonium bromide (AB), (iii) guanidine dihydrogen phosphate (GDP, Scheme 1a), a phosphorus- and nitrogen-containing organophosphate, and (iv) guanylurea methylphosphonate (GUP, Scheme 1b), a phosphorus- and nitrogen-containing organophosphonate with a different P:N ratio to that in (iii).

### 2.2. Sample Preparation

#### 2.2.1. Fire Retardant Treatment

Flax, Flax/PP, and flax/PLA fabrics were treated with aqueous FR solutions using a pad-dry technique to obtain specified fire-retardant element concentrations in the composites as given in Table 1. PP and PLA fibres in sliver form were also treated with FRs to have similar FR pickups as for the fabrics.

#### 2.2.2. Composite Preparation

Eight layered laminates from flax/PP, flax/PLA and FR treated flax/PP, and flax/PLA fabrics were prepared by melt-pressing required layers of each fabric at 180 °C and 40 kg/cm^2^ pressure for 3 min, and then transferring them to a cold press to cool down under pressure (20 kg/cm^2^) to room temperature.

### 2.3. Pyrolysis Combustion Flow Calorimetry (PCFC)

PCFC, Fire Testing Technology Ltd., East Grinstead, UK, was used for flammability assessment of all samples. The heating rate was 1 °C/s to 750 °C in the pyrolysis zone. The pyrolysis was conducted under nitrogen. The combustion temperature was set at 900 °C. The flow was a mixture of O_2_/N_2_ 20/80 cm^3^/min and the sample weight was 3–5 ± 0.5 mg. To obtain small samples of similar sizes for PCFC measurements, fibres were cut to similar length (~2 mm) and composites were crushed into small pieces (~2 mm diameter). Similar sample geometries were also used for TGA work.

### 2.4. Thermogravimetric Analysis

Thermal stabilities of composites and components were studied using SDT 2960 simultaneous DTA-TGA (TA Instruments, Elstree, Hertfordshire, UK) from room temperature to 700 °C at 10 °C/min heating rate under flowing air atmosphere (100 mL/min). The sample size was ~10 mg.

## 3. Results

### 3.1. Pyrolysis Combustion Flow Calorimetry vs. Cone Calorimetry as an Indicator of Flammability

As discussed before, PCFC as opposed to cone calorimetry has been used as an indicator of flammability or fire retardancy of the composites and their components. It is well known that PCFC is not suitable for heterogenous materials such as fibers and in particular surface-treated fibers, where high variance of results can be seen [14]. To address this issue, all samples were cut into very small fibres before testing.

**Figure 1:**
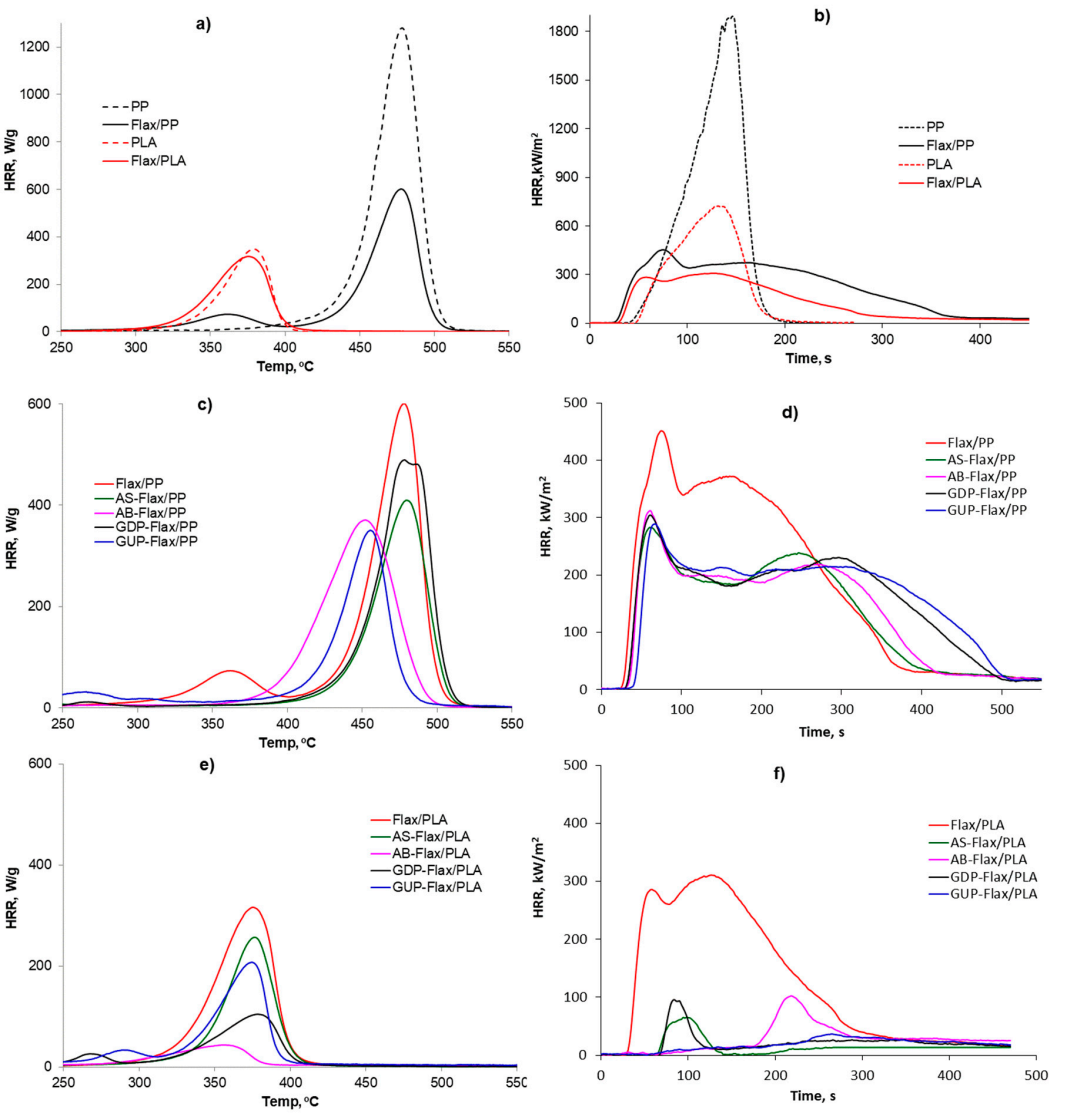
Heat release rate (HRR) versus (**a**,**c**,**e**) temp in pyrolysis flow calorimetry (PCFC) and (**b**,**d**,**f**) time in cone calorimetry at 35 kW/m^2^ for flax/PP and flax PLA composites, and PP and PLA plaques.

PCFC involves controlled pyrolysis of the sample in an inert gas followed by high temperature oxidation of the volatile pyrolysis products. Oxygen consumption theory is used to measure the heat of combustion of the pyrolysis products. The heat release vs. temp curves are obtained, which for all the composites are presented in Figure 1 and for components in different figures in the following sections. The heat release capacity (HRC), defined as the maximum heat release rate divided by the constant heating rate in the test, can be used as a reliable indicator of a polymer’s flammability [13,15,16]. In composites where two peaks were obtained, sums of the peaks as in Equation (1) were calculated and presented.

S = sumHRC = HRC1 + HRC2(1)

Other principal parameters are the temperature at the maximum heat release rate (*T*_max_) and the total heat released; all of these parameters for all the samples are presented in Table 2.

The first step is to determine how PCFC results correlate with cone calorimetry results for those samples on which cone calorimetry can be carried out, i.e., composites and plaques. The heat release rate (HRR) vs. temp (PCFC) and HRR vs. time (Cone) curves for PP, flax/PP, PLA, and flax/PLA composites are presented in Figure 1a,b; the cone results are taken from Ref [11]. While both techniques use oxygen consumption theory for HRR measurement [13,17], they embody two different approaches. In PCFC the sample is heated to 750 °C at a constant heating rate (1 °C/s), in nitrogen, hence pyrolysis is complete, the volatile products then pass into another chamber where oxygen is supplied, and combustion occurs. In cone calorimetry, volatiles are released from the polymeric material, and when the mass of flammable volatiles reaches a critical value, ignition occurs, and the heat released is measured as a function of time. Hence, in PCFC initiation and time-to-peak of heat release are dependent on pyrolysis temperature, whereas in cone calorimetry these are ignition dependent. This difference can be seen more clearly for the PP and PLA results displayed in Figure 1a,b in which both cone calorimetric HRR curves cover the same time range (35–200 s) since PP and PLA have similar TTI (31–34 s). While TTI is dependent on the onset of decomposition temperature of the polymer, for ignition to occur there is a critical mass flux of combustible volatiles. In the case of PCFC however, the HRR peak is between ~320 and 420 °C in PP compared to 420–520 °C in PLA. The relative heights of the peaks, (i.e., HRC or PHRR) for PP and PLA in both tests, however, are similar. For flax/PP, two peaks are seen in both tests, the difference between them being that in PCFC the first peak, which is small, represents pyrolysis of the flax, whilst the second, larger peak arises from PP. In the cone calorimetric traces, the first peak is sharper, whereas the second one is ill-defined and very broad, although the height is comparable to that of the first peak. The first peak represents the ignition of the PP matrix followed by burning of the composite. During this stage the flax fibres start charring, the charred layer acts as a thermal barrier for the underlying polymer, slowing down its burning until the charred layer cracks after which the second peak appears [11]. In flax/PLA, the cone calorimetric traces are similar to those of flax/PP, i.e., two peaks are present, although the first peak is of lower intensity. However, there is only one peak in the PCFC traces. The latter behaviour will be discussed in the following sections as it is important to understand the behaviour of flax. On comparing the PCFC results for flax/PP and flax/PLA, it can be seen that HRC of the former (783 J/g-K) is much higher than the latter (358 J/g-K), which is as expected considering that the HRC of PLA (377 J/g-K) is lower than that of PP (1510 J/g-K), as can be seen from Table 2.

### 3.2. Effect of Fire Retardants on Composites: PCFC vs. Cone Calorimetry vs. Thermal Analysis

The effects of the four fire retardant formulations on the flammabilities of flax/PP and flax/PLA composites studied by PCFC and cone calorimetry are shown in Figure 1c–f. In PCFC of flax/PP, all fire retarded samples show only one peak; the first peak representing pyrolysis of flax in flax/PP is missing, indicating that all the fire retardants have effectively fire retarded the flax component. Reduction in the peak height of the PP component can also be observed, particularly significant reductions being observed for the AB and GUP treated samples. In the case of flax/PLA, all fire retardants reduced the HRC compared to control, the maximum being for the AB followed by GDP treated samples. The cone calorimetric results for these samples have been discussed in detail previously [11].

**Figure 2:**
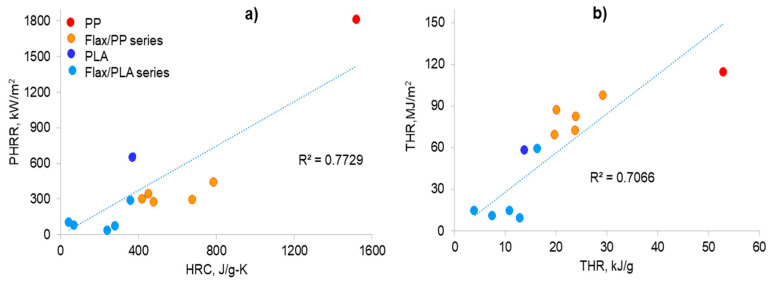
Correlation between PCFC and cone calorimetric results: (**a**) HRC from PCFC versus PHRR from cone calorimetry and (**b**) THR from PCFC versus THR from cone calorimetry for PP, PLA, and composites (both unmodified and fire retarded).

In order to investigate how HRC and THR values from PCFC compare with the corresponding PHRR and THR values from cone calorimetric results, these two sets of parameters for flax/PP and flax/PLA composites and for their fire retarded counterparts are plotted in Figure 2, in which results for PP and PLA plaques are also included. As can be seen from Figure 2 the correlation between HRC and PHRR is poor. It can be seen also that there is not much difference in the variability of results for pure polymers or composites and fire retarded composites. Hence, this variation is not due to FR treatment, but to the technique used to assess FR parameters as has also been demonstrated by other researchers [18,19,20,21]. However, as mentioned before, the purpose of this study is not to assess the flammability of composites and components by PCFC in absolute terms, but to compare the flammabilities of composites with those of their respective components, so that the effects of different fire retardants can be better understood.

In order to investigate the relative effect of each fire retardant on flax/PP and flax/PLA as assessed by both PCFC and cone calorimetry, the percentage changes in all important parameters for all fire retarded samples with respect to those of the respective control (flax/PP or flax/PLA) composites (the latter taken from data presented in [11] are given in Table 3. This table also includes UL-94 results (taken from ref [11])).

While for all fire retarded flax/PLA composites in cone calorimetry, % reduction in PHRR (74–84%) and THR (56–87%) is much higher than for flax/PP (PHRR = 31–37% and THR = 11–29%), in PCFC, except for some samples (AB and GDP treated), the change is similar in both flax/PP and flax/PLA samples, which is surprising. This perhaps is because in cone experiments soon after ignition flax starts to char, the charred layer then slows down burning and hence reduces heat release. All FRs improved char formation as seen from cone results in Table 3, which helped in producing a greater reduction in heat release. Char formation in fire retarded flax/PLA composites is more than in flax/PP composites (see Table 3). On the other hand, in PCFC temperature dependent pyrolysis is very rapid, hence there is less chance of char formation which can act as a thermal insulator for the underlying polymer. As seen from Table 3, in flax/PLA composites more smoke is formed compared to flax/PP composites. Smoke formation is a result of incomplete combustion. This also means that all FRs are changing the decomposition pathway from chain unzipping to hydrolysis of PLA, resulting in, for example, formation of low molecular weight alcohols and acids, producing more smoke. Moreover, smoke producing samples showed worst fit between the two THR values, the exception being the AB treated sample. The latter can be explained on the basis that ammonium bromide mainly acts in the vapour phase and hence oxidation of char does not contribute to THR.

**Figure 3:**
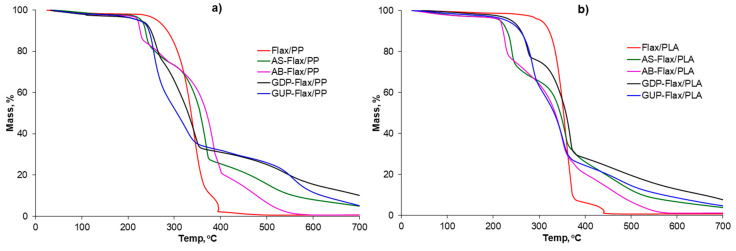
TGA curves of control and FR treated (**a**) flax/PP and (**b**) flax/PLA composites in air.

To understand this better, thermogravimetry of all composites in air atmosphere was performed; the results are shown in Figure 3 and the analysed results are given in Table 4. The flax/PP composite thermally decomposes in two steps. The first step occurs between ~220 and 380 °C leading to 87.8% mass loss with a DTG maximum at 341 °C, representing pure thermal decomposition. In the second step, 10.0% mass loss occurs between 380 and 395 °C, with a DTG maximum at 391 °C, representing oxidation of char formed in the first step. Flax/PP leaves almost no charred residue (only 0.3%) at 700 °C. Flax/PLA composite also displays two mass loss steps, the first step occurs between 252 and 388 °C, with 91.1% mass loss and a DTG maximum at 358 °C, and the second between 388 and 440 °C with 6.3% mass loss and DTG maximum at 436 °C, leaving 0.8% residue. Except for the delayed start of the decomposition in flax/PLA, the mass loss profiles and mass losses in both steps for both composites are similar.

As can be seen from Figure 3, the effect of FRs is similar in both flax/PP and flax/PLA composites. Onset of decomposition (*T*_Onset_) is decreased in all fire retarded samples compared to the respective flax/PP or flax/PLA control samples, the exact value depending upon the FR type. In all cases, the first mass loss representing the thermal decomposition stage in the control samples has changed to a two-step process in fire retarded samples, with overall mass losses less than those of the respective controls (flax/PP or flax/PLA), whereas mass loss in the last (oxidation of the char) stage is more in fire retarded samples than in the control samples. The char yields depend on FR type in both flax/PP and flax/PLA. Since both flax/PP and flax/PLA contain 50% flax, the effect of each FR on this component in both composites should be same, and any differences should be due to the reactivities of the FRs towards the PP or PLA component.

AS (ammonium sulfamate) produces the first (additional) decomposition step in both flax/PP and flax/PLA over a relatively low but similar temperature range (~194–293 °C), the mass loss though is more in flax/PLA (~30%) than in flax/PP (24%), which suggest that AS, apart from catalysing dehydration/decomposition of flax, is also affecting the PLA. The second mass loss step up to ~400 °C occurs with more mass loss in flax/PP (48.6%) than in flax/PLA (40.6%). The mass loss in the char oxidation stage is similar in both composites (22.4%), which suggests that no additional/different char is formed in flax/PLA and flax/PP when AS is present. Ammonium sulfamate is known to hydrolyse the crystalline regions of cellulosic fibres [22]. The reduction of crystallinity decreases the amount of laevoglucosan produced on thermal degradation and consequently the flammability. There is also possibility that water released during dehydration of cellulose would help in decomposition of AS producing, ammonia and sulfuric acid Equation (2), the latter could then provide the condensed-phase FR activity by reacting with components of the composite through a sulfation reaction, particularly with the flax fibre. As a result of sulfation, sulfate ester is formed in the sample, and influences the decomposition of the flax to promote more char formation at the expense of flammable volatiles, as can be seen from the comparatively low mass loss in second decomposition steps of AS-Flax/PP and As-Flax/PLA, and the increase in the char yields in comparison to those of the control, Table 3. This condensed-phase activity results in less smoke production as seen from cone results in Table 3. In the case of flax/PLA there is also the possibility that any water produced during dehydration of the cellulosic part hydrolyses PLA leading to cleavage of ester linkages and hence decomposition [23]. This may explain the greater mass loss in the first stage of flax/PLA decomposition.

(2) $$NH_{4}\left(SO_{3}NH_{2}\right)\overset{H_{2}O,\Delta }{\to }2NH_{3}+H_{2}SO_{4}$$

The AB (ammonium bromide) treated flax/PP and flax/PLA samples also show three mass loss stages. The first step occurs at relatively lower temperatures than in the controls, but over a similar temperature range (~200–245 °C) for both flax/PP and flax/PLA composites. The mass loss though is less in flax/PP (13.1%) than in flax/PLA (21%). The mass loss in the second decomposition step is much higher (64 and 55% for flax/PP and flax/PLA, respectively) than for all other FR treated samples (20–48%), indicating that AB has less effect on the decomposition of polymeric components. In the char oxidation stage though mass loss is less, but similar in both flax/PP and flax/PLA (~18.5%), leaving minimal residue (~1%) at the end. This shows that AB improves the thermal stability of flax/PP and flax/PLA, but the effect is less than that observed for other FRs, especially with respect to char formation. This is because AB generally works as a vapour-phase fire retardant rather than in the condensed phase, mainly owing to the HBr produced from decomposition of AB Equation (3). The vapour-phase activity of AB is supported by the greater smoke formation observed in the cone experiments for AB treated samples (Table 3).

(3) $$NH_{4}Br\overset{\Delta }{\to }NH_{3}+\mathrm{HBr}$$

**Scheme 2:**
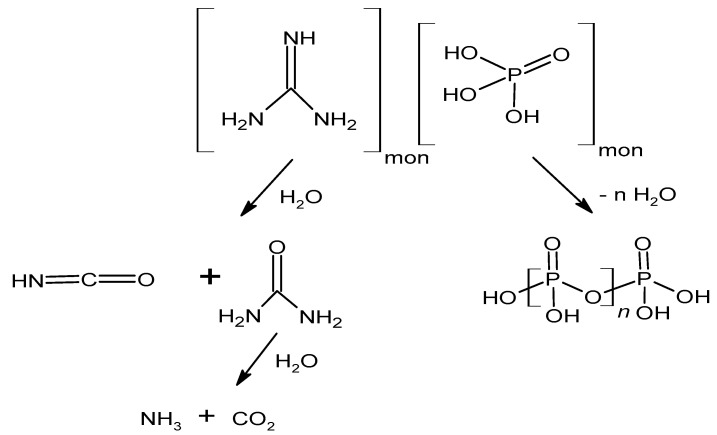
Thermal decomposition of guanidine dihydrogenphosphate (GDP) yielding principally phosphorus acids and anhydrides plus ammonia and carbon dioxide.

GDP (Guanidine dihydrogen phosphate) treated flax/PP and flax/PLA exhibit a first mass loss step between ~200 and 290 °C with a similar mass loss of ~22%, indicating fire-retardant activity in the composites and particularly its reactivity with flax. The second mass loss step is similar in both samples in terms of temperature range (~275–390 °C) and mass loss, which is slightly less in flax/PP (43.0%) than in flax/PLA (47.7%). In the char oxidation stage, the trend is the reverse, i.e., slightly more mass loss (26.9%) in flax/PP than in flax/PLA (20.9%), indicating some effect of GDP on PLA as well. In both cases 5.0–7.6% char residue is left at the end of the experiment, indicating condensed-phase activity of GDP. Phosphorus acids and anhydrides produced from the decomposition of GDP in the first step could react with the composite’s components, particularly flax, through phosphorylation [24] to form phosphate ester which influences the decomposition pathway of the sample to yield more char and less flammable product [25,26]. Guanidine on further heating decomposes into cyanamide derivatives, NH_3_ and, in the presence of water released by condensation of phosphate groups, also CO_2_ (Scheme 2). Moreover, released NH_3_ and CO_2_ may act as blowing agents for the phosphate esters being generated helping in intumescent char formation. Some of the cyanamide derivatives could also be volatile [27] and may also contribute in this way. Phosphorus-nitrogen synergism is a well-known phenomenon [28], in this case, in the presence of nitrogen, the phosphorus acids produced from decomposition of GDP can form P-N bonded intermediates, which are more reactive phosphorylating agents, hence leading to the enhancement in the efficiency in the condensed-phase to improve char formation [29,30].

While phosphorus compounds tend to be less efficient in non-charring PP than charring flax, the effectiveness of phosphorus-based FR, particularly APP, in combination with nitrogen compounds in PP is well known [29,30]. Various proposed mechanisms of action include: (i) the formation of polyphosphoric acid as a surface coating, (ii) the heat sink action of the vaporizing phosphorus compound, (iii) dilution of the combustible pyrolysates by a less combustible vapour and (iv) reduction of melt viscosity to favour a melt drip mode of flame extinction [29] and reference cited within]. In the presence of a char-former, an additional chemical effect of char promotion exists [29], which in this case is the presence of flax char produced by the action of GDP.

In the case of PLA, while a similar mechanism occurs, the released acid could also hydrolyse PLA, leading to cleavage of ester linkages as discussed above with AS.

This therefore results in the higher thermal stabilities of GDP-Flax/PP and GDP-Flax/PLA (Table 4), and the lower flammabilities in comparison to those of the respective control samples (Table 3). Moreover, in comparison to other FR treated flax/PP and flax/PLA, the amount of char formed is higher, supporting a condensed-phase mechanism as discussed above. GDP has little effect on smoke for flax/PP suggesting little or no vapour-phase activity. However, for flax/PLA, both char and smoke are increased (Table 3), suggesting a mixed mechanism.

In the case of GUP (guanylurea methylphosphonate) treated composites, the first step between ~185 and 310 °C is accompanied by highest mass loss in both flax/PP and flax/PLA (44.4 and 36.2%, respectively) among all fire retarded samples. The mass loss in this step is related to the decomposition of the components of the GUP formulation at relatively low temperature due to its relatively low thermal stability and its reaction with flax, and possibly also with PP and PLA. The mass loss in the second step is less (19.8% in flax/PP and 35.1% in flax/PLA) than for all other samples (40–64%,), which indicates less decomposition of the polymer, PP or PLA. The char residue, 3–5%, is higher than those of the AS and AB treated samples, but lower than for the GDP treated ones. GUP starts decomposing at about 180 °C, forming polyguanidine with the gaseous release of NH_3_ and CO_2_, followed by release of polyphosphoric acid [31,32]. The released NH_3_ and CO_2_ act as diluents for combustible gases produced from PP and PLA components of the composites.

**Scheme 3:**
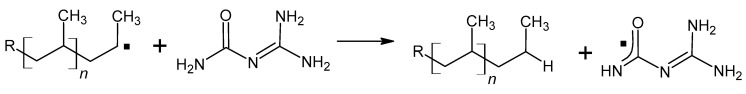
Possible radical inhibition/retardation of PP chain unzipping by reaction with guanylurea.

**Scheme 4:**
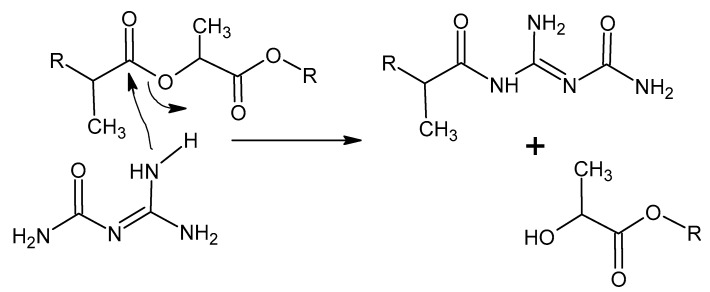
Suggested aminolysis of PLA with guanylurea forming oligomers with guanyl and hydroxyl end groups. Note that further reactions of PLA with the remaining primary and secondary amino groups of guanyl end groups would lead to crosslinking.

The condensed-phase action of GUP due to released phosphorus acids and P-N synergistic action are probably similar to those explained above for GDP. The condensed-phase activity is supported by high char formation in both flax/PP (3.1%) and flax/PLA (4.6%), which is less than with GDP. The vapour-phase activity of GUP by possible radical inhibition/retardation of PP chain unzipping is proposed as in Scheme 3. In case of PLA, aminolysis of PLA with guanylurea forming oligomers with guanyl and hydroxyl end groups could occur as suggested in Scheme 4. Mixed vapour and condensed-phase activity of GUP explains it having the greatest efficiency compared to other FRs as demonstrated by the cone calorimetric and UL-94 results in Table 3. GUP treated samples, however, produce more smoke than all other samples (Table 3), presumably through efficient vapour-phase activity leading to incomplete combustion.

In summary, AS and AB resulted in more mass loss in the first stage and less in second stage in flax/PLA composites compared to flax/PP composite representing some effect of FR decomposition on hydrolysis of PLA, whereas there is similar mass loss in the third (oxidation) stage. GDP and GUP on the other hand had minimal effect on the first stage of decomposition, produced more mass loss in the second stage (arising from an effect on the decomposition of PLA), and less in the third stage in flax/PLA than in flax/PP.

While this information is useful for discussing the mechanism of action of FR type this still does not explain why all FRs are more effective in flax/PLA than flax/PP in UL-94 and cone calorimetric tests (Table 3). Hence, in the next section the effect of each FR on individual components is studied.

### 3.3. Flammability of Components and Effect of Fire Retardants

**Figure 4:**
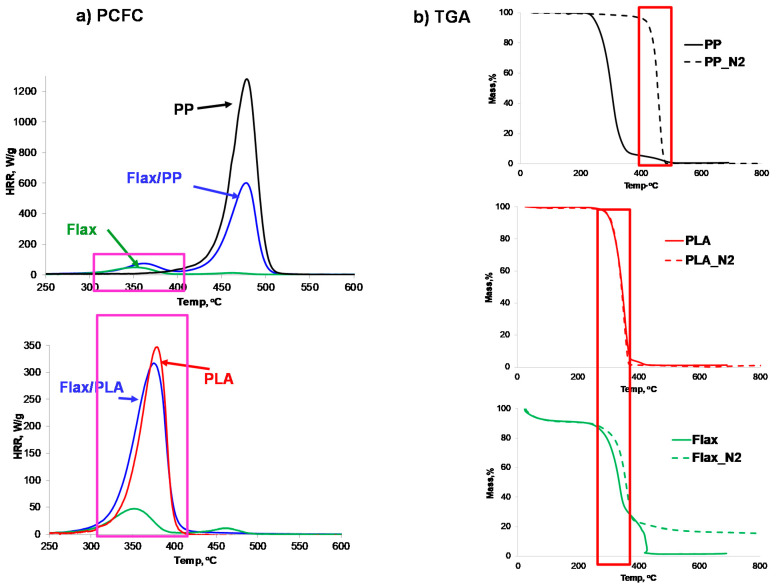
(**a**) PCFC and (**b**) TGA curves in air and nitrogen of PP, PLA, flax, flax/PP, and flax/PLA.

In order to understand the role of each component in a composite, PCFC and TGA in air and nitrogen of PP, PLA, and flax are plotted in Figure 4. The PCFC of composites are also given for comparison. The differences seen previously for temperature ranges of the peaks in PP and PLA polymers and their composites is replicated by decomposition temperature range in TGA under N_2_. The thermal decomposition of PP in air and nitrogen are very different as discussed in detail in the literature [29]. PP undergoes chain scission, demonstrated by a single mass loss step in nitrogen. In air chain scission is catalysed by oxidation of PP at tertiary H giving hydroperoxyl groups which then undergo thermolysis to give hydroxyl and polymeric carboxy radicals. The hydroxyl radicals catalyse further oxidation. The carboxy radicals undergo rearrangement and scission giving carbonyl-ended chains and polymeric radicals capable of unzipping to give volatile oligomeric fragments [29]. There is a small amount of crosslinking also which leads to incipient char formation (see Table 5). The higher decomposition temperature range in nitrogen explains the long time-to-PHRR in PCFC. PLA thermally decomposes in a set of sequential and parallel reactions consisting of (i) intra- and intermolecular ester exchange producing lactide and cyclic oligomers, (ii) cis-eliminations producing acrylic acid and acyclic oligomers and (iii) radical and concerted reactions producing acetaldehyde, carbon monoxide, and carbon dioxide; no char is formed [33].

Flax, a cellulosic fibre with 2–3% lignin and ~2% pectin [2] is a char forming polymer. Cellulose undergoes two types of decomposition reactions depending upon the heating condition. At temperatures between 200 and 280 °C, dehydration and depolymerisation reactions occur leading to formation of dehydrocellulose, which further decomposes to form char and volatile products. At the higher temperature, i.e., 280–340 °C, it decomposes to form a liquid intermediate product laevoglucosan which subsequently decomposes to produce highly flammable volatiles, and a little charred residue. Hence if cellulose is heated slowly, the first type of reaction is favoured, which leads to high char formation [8]. The lignin (a three dimensional and highly crosslinked aromatic structure) present in the flax (~2–3%) also helps in char formation [2]. The TGA curve for flax, as expected, shows a two stage decomposition in air and one step in N_2_. As can be seen from Table 5 and Figure 4, the temperature range of decomposition of flax is similar to that of PLA; hence in the case of PCFC there is only one peak, where both PLA and flax are undergoing combustion as opposed to that of flax/PP where the flax peak is seen first and then that of PP.

To have a better understanding of whether the FR reacts only with flax or with PP and PLA as well, flax fabric, PP and PLA fibres (in sliver form) were treated with different FRs to have a similar FR pickup as in the above section (Table 1).

#### 3.3.1. Effect of AS on Components

**Figure 5:**
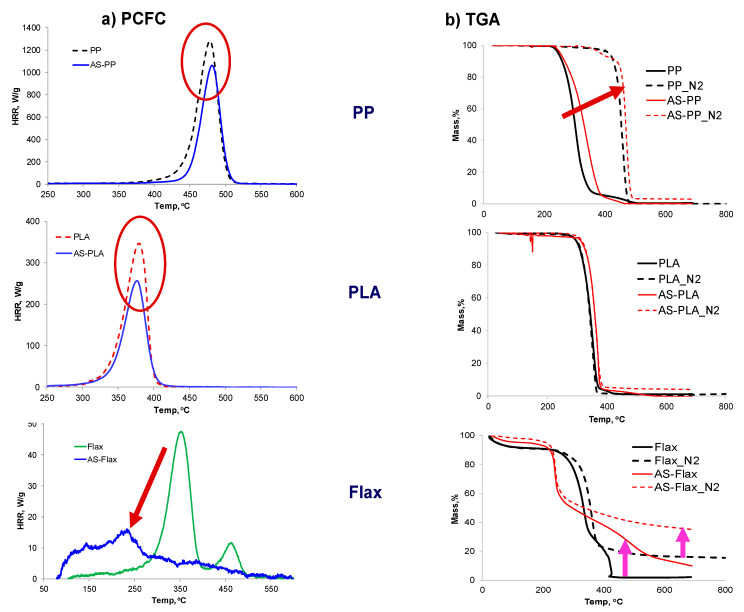
(**a**) PCFC and (**b**) TGA curves (in air and nitrogen) of ammonium sulfamate (AS) treated PP, PLA, flax, flax/PP, and flax/PLA.

The PCFC and TGA curves showing the effects of AS on flax, PP and PLA are given in Figure 5. AS reduces PHRR of PP by 28% with respect to the virgin PP. TGA results show that in air the thermal stability of the polymer is increased, indicating perhaps that some radicals are produced from the FR, which help in terminating the radical products of chain scission. Thermal stability in nitrogen is also increased, but the effect is minimal. In PLA, AS reduced HRC of PLA by 26% (Figure 5), which indicates some action of AS on PLA. The TGA curves of AS treated PLA in both air and N_2_ are similar to those of the respective PLA curves, indicating no interaction, although 4% char residue is left in N_2_. This similar TGA behaviour of PLA and fire retarded polymer in both air and nitrogen atmospheres has also been reported in the literature [34], indicating that the FR does not react with PLA, but changes its mode of decomposition. This mode of decomposition may involve hydrolysis of the PLA catalysed by sulfuric acid formed from the AS leading to the production of -OH and -COOH terminated oligomeric products and thus altering the decomposition pathway. AS is very effective on flax with 65% reduction in HRC. The TGA curves both in air and nitrogen indicate the thermal stability of the AS treated flax, leading to 35% char yield in nitrogen. The condensed-phase activity of AS in flax has already been discussed in the previous section.

#### 3.3.2. Effect of AB on Components of Composites

**Figure 6:**
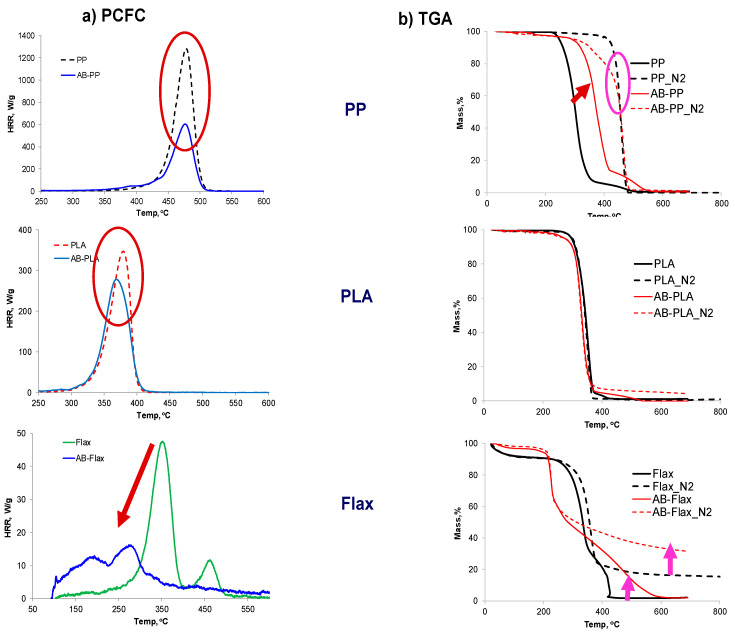
(**a**) PCFC and (**b**) TGA curves (in air and nitrogen) of ammonium bromide (AB) treated PP, PLA, flax, flax/PP, and flax/PLA.

AB is very effective in reducing the HRC of PP, where 57% reduction could be obtained. The TGA curve in air shows that AB treated PP has significantly improved thermal stability in terms of less mass loss. Thermal decomposition of AB produces HBr, which acts mainly in vapour-phase; Br· radicals from HBr react with ·OH radicals produced in the oxidative degradation of PP, retarding the progress of the catalysed oxidative degradation of PP, hence reduced weight loss as compared to PP when TGA is carried out in air. However, there seems to be little effect of Br· radicals on decomposition of PP under nitrogen. In PLA while there is 23% reduction in HRC in PCFC, there is not much difference in TGA behaviour in air or nitrogen although 4.5% char is formed. HBr, an acid, may catalyse hydrolysis of PLA leading, as with AS, to the formation of -OH and -COOH terminated oligomeric fragments. AB in flax is very effective, where it is working in both vapour- and condensed-phase. Any moisture or water vapour released during dehydration of cellulose will tend to hydrolyse AB, producing HBr, which, acting as a Lewis acid, will catalyse dehydration of the flax, leading to much enhanced char formation as shown in Figure 6.

#### 3.3.3. Effect of GDP on Components

**Figure 7:**
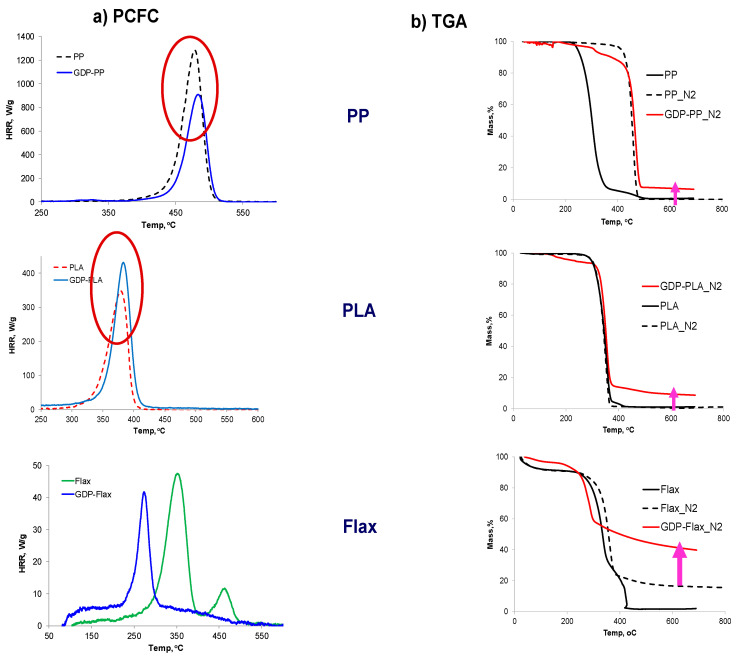
(**a**) PCFC and (**b**) TGA curves (in nitrogen) of GDP treated PP, PLA, flax, flax/PP, and flax/PLA.

The effect of GDP on PP seen from Figure 7 indicates about a 30% reduction in HRC, whereas in PLA there is a small increase and in flax very little effect (ca. 10% reduction). The TGA results in nitrogen though show that in PP, while GDP causes mass loss at a lower temperature than in PP, it does not affect the mass loss over the main decomposition stage, but leads to 6.5% char formation. In the case of PLA, the effect is also similar: start of mass loss at a lower temperature, but leaving more char residue, 8.7%. The char forming ability of GDP in flax is very pronounced with ca. 40% char left at the end of the experiment. Overall, it could be suggested that these results are consistent with discussion in the previous section that GDP has a mainly condensed-phase (char promoting) effect.

#### 3.3.4. Effect of GUP on Components

**Figure 8:**
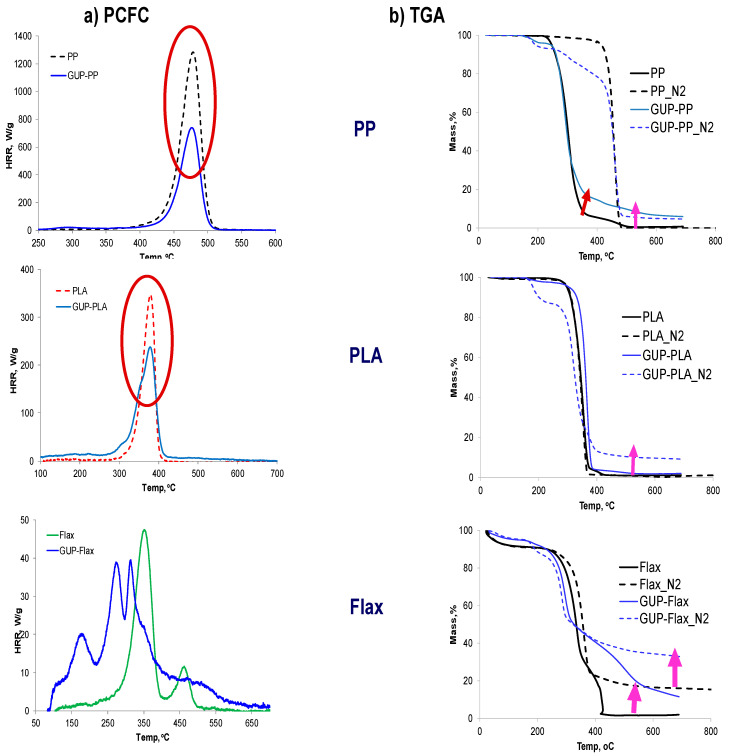
(**a**) PCFC and (**b**) TGA curves (in air and nitrogen) of GUP treated PP, PLA, flax, flax/PP, and flax/PLA.

GUP, guanylurea methylphosphonate, has a significant effect on reducing the combustibility of PP as well as of PLA, as seen from the significant reduction in HRC of 44% in PP and 27% in PLA (Table 2, Figure 8). The TGA curves of GUP-PP in air and nitrogen in Figure 8 show that while the mass loss starts at lower temperature than in PP alone, GUP does not have much effect on the overall decomposition step; however, some char is formed (6.1% in air, 4.7% in nitrogen). This indicates that GUP works in both the condensed- and vapour-phases. GUP possibly acts as a radical inhibitor/retarder of PP chain unzipping (see Scheme 3), forming more char in air. However, any previously published evidence for this mode of action of GUP could not be located in literature. A similar effect is seen for PLA, in which char formation is increased in nitrogen to 9.2%. In PLA, GUP probably reacts with ester links via aminolysis, forming more thermally stable intermediates (See Scheme 4) from which char more readily forms. A similar reaction may be involved also in the condensed-phase fire retardation of PLA with GDP. The concurrent vapour-phase activity of GUP (dilution of combustible volatiles by released NH_3_ and CO_2_) also helps in reducing combustibility.

There is no significant effect on the combustibility of flax seen from PCFC results, though char formation is increased in TGA experiments. This indicates that in flax, FR action is primarily in the condensed-phase.

## 4. Discussion

In comparison to PP, which undergoes decomposition through chain scission to give just hydrocarbon fragments with no char residue, PLA contains oxygen in its chemical structure providing pathways to the formation of decomposition products that are less flammable or burn with lower heat release. Moreover, PLA is extremely sensitive to hydrolysis, leading to the scission of ester bonds and reduction of the molar mass [35], which can contribute to lowering the flammability [23]. The lower flammability of PLA compared with that of PP is demonstrated by its slightly higher LOI (PLA = 19.2; PP = 18.0) [36]; 75% lower HRC and 73% lower THR than PP from PCFC test (Table 2); and 63% lower PHRR and 50% lower THR in cone experiments [36]. Hence, the flammability of flax/PLA composite in terms of heat release in cone calorimetry is much lower than that of flax/PP (Figure 1).

In general, FRs acting in the vapour-phase are more effective in reducing the flammability of PP as these affect the oxidation catalysed chain-scission reactions. On the other hand, FRs that act solely or mainly in the condensed-phase, namely AS, GDP, and GUP, are more effective in PLA. These FRs acid catalyse the hydrolysis of PLA via release of sulfuric (AS) or phosphorus acids (GDP and GUP) and, in the case of GDP and GUP, probably react also via aminolysis with PLA, leading to the formation of, for example, alcohol-, acid-, or amide-terminated oligomers, which are less flammable, and play some role in char formation.

In the case of flax/PLA composites, since the decomposition temperature range of PLA is similar to that of flax, any water vapour released during dehydration/early stages of decomposition of flax will hydrolyse PLA, changing its decomposition pathway. FRs acting in the condensed-phase, in particular phosphorus-based ones, catalyse dehydration reactions of cellulose, which in turn also have an effect on the hydrolysis of PLA. This is shown by similar TGA curves of fire retarded PLA samples as the control, but increased char yield, even though all these samples were rated V-0 in UL-94 tests.

The effectiveness of FRs on flax/PLA compared to flax/PP is more pronounced in cone experiments (Table 3) than in PCFC (Table 2), which is because in the former, soon after ignition the flax starts charring, the charred layer then slows down burning, effecting hydrolysis or aminolysis of PLA, hence reducing heat release. TTI in cone tests for flax/PP and flax/PLA samples increased only with those FRs, which function only or mainly in the vapour-phase, i.e AB and GUP treated ones (Table 3). All FRs decreased PHRR in both flax/PP and flax/PLA, but GUP is the only one which gave rise to a V-0 UL-94 rating in flax/PP, which could be due to it having both vapour- and condensed-phase activity.

The greater impairment in the mechanical properties of all FR treated flax/PLA samples compared with the corresponding flax/PP samples, observed previously [11,12] can also be explained as being due to hydrolysis or aminolysis of PLA during the processing stage.

## 5. Conclusions

This study has enabled understanding the reasons for (i) the lower flammability of flax/PLA composites compared to flax/PP and (ii) the greater effectiveness of fire retardants in flax/PLA than in flax/PP. While PP and PLA are similarly ignitable and burn in a UL-94 test, PLA shows much reduced PHRR and THR (*Ca.* 50–60% compared with PP) in cone calorimetric testing. This behaviour is replicated in flax/PP and flax/PLA composites, i.e., fails in UL-94 tests and similar rates of burning in horizontal mode for both types of composite [11], but much reduced PHRR and THR in flax/PLA. This is due to the pure hydrocarbon structure of PP, decomposing into readily flammable species, whereas in PLA oxygen-containing decomposition products burn with less heat output. All fire retardants chosen based on their well-known chemical/physical interaction during thermal decomposition of cellulosic fibres, effectively act on flax fibres, but have no chemical interaction with PP and relatively little with PLA. While their main action is physical, namely dilution of combustible gases by producing non-combustible gases or encapsulating the decomposing polymer in the intumescing char, in the case of PLA (with the probable exception of AB) they catalyse hydrolysis or react via aminolysis (in the case of GDP and GUP) leading to alcohol-, acid- and amide-terminated intermediates and reduction of molar mass, which results in lower flammability. This explains not only their greater effectiveness in reducing the flammability of flax/PLA compared with flax/PP, but also the reason for the impairment of mechanical properties of the composites.

Overall it can be concluded that while the use of conventional FR formulations and techniques for fire retarding biobased composites is a good approach, commercial formulations need to be modified to avoid the hydrolysis of, or some other reaction with, PLA at processing stages.

## Figures and Tables

**Table 1:** Details of flax/PP, flax/PLA, flax fabrics and PP, PLA fibres treated with different fire retardant formulations.

Sample	FR Content on Fabric (%)	P Content (%)	N Content (%)	S Content (%)	Br Content (%)
AS-Flax/PP; AS-Flax; AS-PP	10.9 ± 1.7	-	1.1 ± 0.2	0.8 ± 0.1	-
AB-Flax/PP; AB-Flax; AB-PP	12.1 ± 0.8	-	1.4 ± 0.1	-	1.9 ± 0.1
GDP-Flax/PP; GDP-Flax; GDP-PP	10.6 ± 0.9	1.0 ± 0.1	1.1 ± 0.1	-	-
GUP-Flax/PP; GUP-Flax; GUP-PP	11.2 ± 2.1	1.0 ± 0.2	1.6 ± 0.3	-	-
AS-Flax/PLA; AS-Flax; AS-PLA	10.1 ± 1.1	-	1.0 ± 0.1	0.8 ± 0.1	-
AB-Flax/PLA; AB-Flax; AB-PLA	13.2 ± 1.2	-	1.5 ± 0.1	-	2.0 ± 0.2
GDP-Flax/PLA; GDP-Flax; GDP-PLA	9.7 ± 0.5	0.9 ± 0.1	1.0 ± 0.1	-	-
GUP-Flax/PLA; GUP-Flax; GUP-PLA	9.9 ± 1.4	0.8 ± 0.1	1.4 ± 0.2	-	-

**Table 2:** PCFC results of unmodified and fire retarded Flax, PP, PLA, Flax/PP, and Flax/PLA fibres.

FR Treat	Flax/PP			Flax			PP			Flax/PLA			PLA		
	HRC	PT	THR	HRC	PT	THR	HRC	PT	THR	HRC	PT	THR	HRC	PT	THR
-	783 ^S^	312; 478	29.2	52	351	3.9	1510	478	52.9	358	375	16.3	377	380	13.8
AS	482	479	19.5	18	233	3.8	1241	481	39.4	278	376	10.9	280	376	11.0
AB	430	452	23.6	19	275	4.0	646	476	28.5	66 ^S^	357	4.1	290	369	13.5
GDP	583	478	23.6	49	273	3.7	1054	484	38.6	155 ^S^	379	7.1	495	383	19.7
GUP	423	455	20.1	47	314	8.2	848	477	33.3	283 ^S^	374	12.5	274	370; 378	17.9

Note: HRC = Heat release capacity ((J/g-K); PT = Peak temp. (°C); THR = total heat release (kJ/g); HRC value with ‘s’ denotes sumHRC.

**Table 3:** Percentage change in flammability parameters w.r.t those of the respective control samples.

FR Treatment	PCFC		Cone Calorimetry					UL-94
	HRC (%)	THR (%)	TTI (s)	PHRR (%)	THR (%)	TSR (%)	Char (%)	Rating
AS-Flax/PP	−38	−33	+5	−37	−29	−10	+12	Fail
AB-Flax/PP	−45	−19	+7	−31	−26	+40	+11	Fail
GDP-Flax/PP	−26	−19	+5	−31	−15	+4	+17	Fail
GUP-Flax/PP	−46	−31	+15	−34	−11	+79	+13	V-0
AS-Flax/PLA	−22	−33	+31	−74	−74	+13	+26	V-0
AB-Flax/PLA	−82	−75	+156	−56	−74	+133	+20	V-0
GDP-Flax/PLA	−57	−56	+39	−72	−81	+163	+32	V-0
GUP-Flax/PLA	−21	−23	+226	−87	−84	+289	+20	V-0

**Table 4:** TGA and DTG results of unmodified and fire retarded flax/PP and flax/PLA composites in air.

FR Treatment	Flax/PP					Flax/PLA				
	Mass Loss Stages			DTG Maxima (°C)	Char at 700 °C (%)	Mass Loss Stages			DTG Maxima (°C)	Char at 700 °C (%)
	Stage	Temp Range (°C)	Mass Loss (%)			Stage	Temp Range (°C)	Mass Loss (%)		
-	I	219 *–380	87.8	341	0.3	I	252 *–388	91.1	358	0.8
	II	380–395	10.0	391		II	388–440	6.3	436	
AS	I	194 *–293	23.8	239	2.9	I	201 *–290	29.6	240	3.9
	II	293–385	48.6	368		II	290–412	40.6	356	
	III	385–589	22.4	497 ^s^		III	412–565	22.2	463 ^s^	
AB	I	209 *–241	13.1	225	0.8	I	206 *–246	21.0	225	1.1
	II	241–412	64.2	384		II	246–405	55.4	349	
	III	412–601	18.7	471 ^s^		III	405–590	18.5	458 ^(s)^	
GDP	I	200 *–276	22.2	261	5.1	I	199 *–290	21.5	273	7.6
	II	276–376	43.0	344		II	290–392	47.7	366	
	III	376–633	26.1	537 ^s^		III	392–614	20.9	467 ^s^	
GUP	I	186 *–299	44.4	256	3.1	I	192 *–310	36.2	286	4.6
	II	299–381	19.8	324		II	310–393	35.1	351	
	III	381–630	29.9	557 ^s^		III	393–623	21.3	475 ^s^	

Note: * = Onset of decomposition temperature. There is small mass loss (~3%) from room temperature (RT) to the onset of decomposition temperature, corresponding to the volatilisation of moisture in the samples. s = represents small peak.

**Table 5:** TGA, and DTG results of unmodified and fire retarded Flax, PP, PLA, Flax/PP, and Flax/PLA fibres in air and nitrogen (given in parentheses).

FR Treat-Ment	Flax			PP			PLA		
	T_Onset_ # (°C)	DTG Maxima (°C)	Char at 700 °C (%)	T_Onset_ * (°C)	DTG Maxima (°C)	Char at 700 °C (%)	T_Onset_ * (°C)	DTG Maxima (°C)	Char at 700 °C (%)
-	232 [250]	338, 405 ^s^ [365]	2.0 [16.0]	227 [375]	306, 472 ^s^ [463]	0 [0]	280 [280]	355, 410 ^s^ [354]	0.7 [0.7]
AS	120 [160]	241, 502 ^s^ [243]	9.8 [35.2]	242 [355]	346, 508 ^s^ [383 ^s^, 473]	0 [3.2]	205 [275]	370 [366]	0 [4.0]
AB	162 [162]	226, 260 ^s^, 478 [227]	1.7 [32.0]	198 [198]	373, 518 ^s^ [362 ^s^, 465]	0.5 [1.5]	198 [198]	334, 548 ^s^ [332]	0 [4.5]
GDP	NT [165]	NT [206 ^s^, 290]	NT [39.8]	NT [223]	NT [315 ^s^, 472]	NT [6.5]	NT [153]	NT [356]	NT [8.7]
GUP	152 [152]	188 ^s^, 298, 501 ^s^ [182 ^s^, 286, 370 ^s^]	11.5 [33.0]	174 [174]	183 ^s^, 295, 422 ^s^ [188 ^s^, 300 ^s^, 460]	6.1 [4.7]	203 [165]	368 [182 ^s^, 322, 380 ^s^]	2.0 [9.2]

Note: * = Onset of decomposition temperature (if not clear onset, taken where 2% mass loss occurred; # = in case of flax there was about 10% mass loss prior to T_Onset_, in all FR treated samples there was 3–4% mass loss (taken when mass loss starts after plateau; s = represents small peak. NT = not tested.

## References

[1] Peças P., Carvalho H., Salman H., Leite M. (2018). Natural Fibre Composites and Their Applications: A Review. J. Compos. Sci..

[2] Kandola B.K., John M.J., Thomas S. (2012). Fire Retardant Natural Fibre Composites for High Performance Applications. Natural Polymer: Composite.

[3] Kandola B.K., Mistik S.I., Pornwannachai W., Anand S.C. (2018). Natural Fibre-Reinforced Thermoplastic Composites from Woven-Nonwoven Textile Preforms: Mechanical and Fire Performance Study. Comp. Part A Appl. Sci..

[4] Le Bras M., Duquesne S., Fois M., Grisel M., Poutch F. (2005). Intumescent Polypropylene/Flax Blends: A Preliminary Study. Polym. Deg. Stab..

[5] Schartel B., Braun U., Schwarz U., Reinemann S. (2003). Fire Retardancy of Polypropylene/Flax Blends. Polymer.

[6] Kandola B.K., Anand S.C., Mistik S.I., Pornwannachai W., Mottershead B. Melt Dripping and Flammability Behaviour of Biodegradable Natural Fibre-Reinforced Thermoplastic Composites. Proceedings of the 15th European Conference on Composite Materials (ECCM15).

[7] Helwig M., Paukszt D. (2000). Flammability of Composites Based on Polypropylene and Flax Fibres. Mol. Cryst. Liq. Cryst..

[8] Kandola B.K., Horrocks A.R., Price D., Coleman G.V. (1996). Flame Retardant Treatments of Cellulose and Their Influence on the Mechanism of Cellulose Pyrolysis. J. Macromol. Sci. Rev. Macromol. Chem. Phys..

[9] Hapuarachchi T.D., Peijs T. (2010). Multiwalled Carbon nanotubes and Sepiolite Nanoclays as Flame Retardants for Polylactide and its Natural Fibre Reinforced Composites. Comp. Part A Appl. Sci..

[10] Dorez G., Taguet A., Ferry L., Lopez-Cuesta J.M. (2013). Thermal and Fire Behaviour of Natural Fibers/PBS Biocomposites. Polym. Deg. Stab..

[11] Pornwannachai W., Ebdon J.R., Kandola B.K. (2018). Fire-Resistant Natural Fibre-Reinforced Composites from Flame Retarded Textiles. Polym. Deg. Stab..

[12] Pornwannachai W., Ebdon J.R., Kandola B.K. (2019). Fire-Resistant Flax—Reinforced Polypropylene/Polylactic Acid Composites with Optimised Fire and Mechanical Performances. J. Thermoplas. Comp. Mat..

[13] Lyon R.E., Walters R.N. (2004). Pyrolysis Combustion Flow Calorimetry. J. Anal. Appl. Pyrol..

[14] Yang C.Q., He Q. (2012). Textile Heat Release Properties Measured by Microscale Combustion Calorimetry: Experimental Repeatability. Fire Mater..

[15] Sonnier R., Vahabi H., Ferry L., Lopez-Cuesta J.-M., Wilkie C.A., Morgan A.B., Nelson G.L. (2012). Pyrolysis-Combustion Flow Calorimetry: A Powerful Tool to Evaluate the Flame Retardancy of Polymers. Fire and Polymers VI: New Advances in Flame Retardant Chemistry and Science.

[16] Schartel B., Pawlowski K.H., Lyon R.E. (2007). Pyrolysis Combustion Flow Calorimeter: A Tool to Assess Flame Retarded PC/ABS Materials?. Thermochim. Acta.

[17] (1993). ISO 5660-1:1993. Fire Tests on Building Materials and Structures—Part 15: Method for Measuring the Rate of Heat Release of Products.

[18] Lu H., Wilkie C.A. (2010). Synergistic Effect of Carbon Nanotubes and Decabromodiphenyl Oxide/Sb_2_O_3_ in Improving the Flame Retardancy of Polystyrene. Polym. Deg. Stab..

[19] Cogen J.M., Lin T.S., Lyon R.E. (2009). Correlations Between Pyrolysis Combustion Flow Calorimetry and Conventional Flammability Tests with Halogen-Free Flame Retardant Polyolefin Compounds. Fire Mater..

[20] Lyon R.E., Takemori M.T., Safronava N., Stoliarov S.I., Walters R.N. (2009). A Molecular Basis for Polymer Flammability. Polymer.

[21] Lyon R.E., Walters R.N., Stoliarov S.I. (2007). Screening Flame Retardants for Plastics Using Microscale Combustion Calorimetry. Polym. Eng. Sci..

[22] Lewin M. (2015). Unsolved Problems and Unanswered Questions in Flame Retardance of Polymers. Polym. Deg. Stab..

[23] Lesaffre N., Bellayer S., Fontaine G., Jimenez M., Bourbigot S. (2016). Revealing the Impact of Ageing on a Flame Retarded PLA. Polym. Deg. Stab..

[24] Fukushima K., Takai Y., Kanayama A., Ono S., Yoshimura T., Morita H., Tsukurimichi E., Shimasaki C. (1996). Thermal Behavior of Guanidinium Dihydrogenphosphate and Diguanidinium Hydrogenphosphate. Nippon Kagaku Kaishi.

[25] Lewin M., Sello S.B., Lewin M., Atlas S.M., Pearce E.M. (1975). Technology and Test Methods of Fireproofing of Cellulosics. Fire retardant Polymeric Materials.

[26] Lewin M., Le Bras M., Camino G., Bourbigot S., Delobel R. (1997). Physical and Chemical Mechanism of Fire Retarding of Polymers. Fire Retardancy of Polymers: The Use of Intumescence.

[27] Jin F., Xia Y., Mao Z., Ding Y., Guan Y., Zheng A. (2014). Special Spherical Shell-shaped Foam Deriving from Guanidine Phosphate—Pentaerythritol System and its Intumescent Fire Retardant Effects on Polypropylene. Polym. Deg. Stab..

[28] Gaan S., Sun G., Hutches K., Engelhard M.H. (2008). Effect of Nitrogen Additives on Flame Retardant Action of Tributyl Phosphate: Phosphorus-Nitrogen Synergism. Polym. Deg. Stab..

[29] Zhang S., Horrocks A.R. (2003). A Review of Fire Retardant Polypropylene Fibres. Prog. Polym. Sci..

[30] Jiang W., Hao J., Han Z. (2012). Study on the Thermal Degradation of Mixtures of Ammonium Polyphosphate and a Novel Caged Bicyclic Phosphate and Their Fire Retardant Effect in Polypropylene. Polym. Deg. Stab..

[31] Wang Q., Li J., Winandy J.E. (2004). Chemical Mechanism of Fire Retardance of Boric Acid on Wood. Wood Sci. Technol..

[32] Li H., Chen M.-L., Lv H.-F., Yang F., Yu L.-L., Fei B.-H., Ma X.-X. (2018). Effects of Guanylurea Phosphate Treatment on the Performance of Decorative Bamboo Filament. BioResources.

[33] Kopinke F.D., Remmler M., Mackenzie K., Moder M., Wachsen O. (1996). Thermal Decomposition of Biodegradable Polyesters—II. Poly(lactic acid). Polym. Deg. Stab..

[34] Yan Y., Gu X., Li L., Li H., Sun J., Zhang S. (2017). Preparation and Characterization of Intumescent Flame Retardant Biodegradable Poly(lactic acid) Nanocomposites Based on Sulfamic Acid Intercalated Layered Double Hydroxides. Fibers Polym..

[35] Zhao C.X., Liu Y., Wang D.Y., Wang D.L., Wang Y.Z. (2008). Synergistic Effect of Ammonium Polyphosphate and Layered Double Hydroxide on Flame Retardant Properties of Poly(vinyl alcohol). Polym. Deg. Stab..

[36] Pornwannachai W. (2015). Flame Retardant Natural Fibre Composites for High Performance Applications. Ph.D. Thesis.

